# Management der Hyperglykämie in der Schwangerschaft (Update 2026)

**DOI:** 10.1007/s00508-025-02695-4

**Published:** 2026-04-30

**Authors:** Alexandra Kautzky-Willer, Yvonne Winhofer, Michael Leutner, Herbert Kiss, Veronica Falcone, Tina Linder, Angelika Berger, Lukas Wisgrill, Monika Lechleitner, Raimund Weitgasser, Simone Huber, Jürgen Harreiter

**Affiliations:** 1 Klinische Abteilung für Endokrinologie und Stoffwechsel, Universitätsklinik für Innere Medizin III, Medizinische Universität Wien, Wien, Österreich; 2 Klinische Abteilung für Geburtshilfe und feto-maternale Medizin, Universitätsklinik für Frauenheilkunde, Medizinische Universität Wien, Wien, Österreich; 3 Klinische Abteilung für Neonatologie, Pädiatrische Intensivmedizin und Neuropädiatrie, Universitätsklinik für Kinder- und Jugendheilkunde, Comprehensive Center for Pediatrics, Medizinische Universität Wien, Wien, Österreich; 4 Avomed-Arbeitskreis für Vorsorgemedizin und Gesundheitsförderung Tirol, Innsbruck, Österreich; 5 Kompetenzzentrum Diabetes, Mavie Med Privatklinik Wehrle-Diakonissen, Salzburg, Österreich; 6 1. Medizinische Abteilung mit Diabetologie, Endokrinologie und Nephrologie, Klinik Landstraße, Wiener Gesundheitsverbund, Wien, Österreich; 7 Abteilung für Innere Medizin mit Palliative Care, Landesklinikum Scheibbs, Scheibbs, Österreich

**Keywords:** Gestationsdiabetes, Diabetische Fetopathie, Präkonzeptioneller Diabetes, Diabetische Embryopathie, Perinatale Morbidität, Gestational diabetes mellitus, Diabetic fetopathy, Preconception diabetes, Diabetic embyropathy, Perinatal morbidity

## Abstract

Hyperglykämie in der Schwangerschaft ist mit erhöhter mütterlicher und fetaler Morbidität sowie langfristigen Risiken für Mutter und Kind assoziiert. Unsere aktuelle Leitlinie fasst erstmals Gestationsdiabetes (GDM) und präkonzeptionellen Diabetes in einer gemeinsamen Leitlinie zusammen. Während die Besonderheiten der einzelnen Diabetesformen getrennt dargestellt werden, gelten Empfehlungen zu Lebensstil, Glukosemonitoring und Pharmakotherapie für alle Formen der Hyperglykämie in der Schwangerschaft. Frauen mit in der Frühschwangerschaft diagnostiziertem Diabetes gelten als Schwangere mit manifestem Diabetes, während GDM üblicherweise zwischen der 24. und 28. Schwangerschaftswoche mittels oGTT diagnostiziert wird, bei Hochrisikopatientinnen auch früher. Eine Neuerung ist die Diskussion eigener Diagnosekriterien für einen frühen GDM. Zentrale Therapieelemente sind Ernährungsberatung, regelmäßige Blutzuckerselbstkontrollen und körperliche Aktivität; bei unzureichender Stoffwechselkontrolle ist Insulin die Therapie der Wahl. Bei präkonzeptionellem Diabetes sind Schwangerschaftsplanung, präkonzeptionelle Stoffwechseloptimierung sowie eine engmaschige Betreuung essenziell. Technische Fortschritte im kontinuierlichen Glukosemonitoring, in der Pumpentherapie und bei AID-Systemen gewinnen auch in der Schwangerschaft zunehmend an Bedeutung. Postpartal wird bei Frauen mit GDM ein oGTT nach 4 bis 12 Wochen empfohlen, bei Normalbefund mit Nachsorgeuntersuchungen alle 1 bis 3 Jahre. Alle Betroffenen sollen über ihr erhöhtes Risiko für Typ-2-Diabetes und kardiovaskuläre Erkrankungen aufgeklärt werden. Stillen wird ausdrücklich empfohlen. Kinder von Müttern mit GDM oder Diabetes sollten aufgrund eines erhöhten Risikos für Adipositas und Entwicklungsauffälligkeiten langfristig nachbetreut werden.

## Abstract (en)

Hyperglycemia in pregnancy is associated with increased maternal and fetal morbidity and long-term health risks for both mother and child. For the first time our current guidelines integrate gestational diabetes mellitus (GDM) and preconception diabetes into joint guidelines. While the characteristics of individual diabetes subtypes are addressed separately, recommendations regarding lifestyle, glucose monitoring and pharmacotherapy apply to all forms of hyperglycemia during pregnancy. Women with diabetes diagnosed early in pregnancy are classified as pregnant women with overt diabetes, whereas GDM is usually identified by oral glucose tolerance testing (oGTT) between 24 and 28 weeks gestation, or earlier in high-risk patients. A novel aspect is the consideration of specific diagnostic criteria for early GDM. Key management strategies include nutritional counselling, regular self-monitoring of blood glucose and physical activity, with insulin as the treatment of choice if glycemic targets are not achieved. In women with preconception diabetes, pregnancy planning, preconception metabolic optimization and close interdisciplinary care are essential. Technical advances in continuous glucose monitoring, insulin pump therapy and automated insulin delivery (AID) systems are becoming increasingly more relevant in pregnancy. Postpartum, women with GDM should undergo an oGTT 4–12 weeks after delivery, with follow-up screening every 1–3 years if results are normal. All affected women should be informed about their elevated risk of type 2 diabetes and cardiovascular diseases. Breastfeeding is strongly recommended. Children of mothers with GDM or diabetes require long-term follow-up due to an increased risk of obesity and developmental disorders.

Trotz Fortschritten im Therapiemanagement bestehen bei Frauen mit Hyperglykämie in der Schwangerschaft weiterhin erhöhte Risiken für maternale und neonatale Komplikationen sowie eine gesteigerte perinatale Mortalität. Hauptursachen sind eine unzureichende präkonzeptionelle Betreuung und eine nicht optimierte glykämische Kontrolle zu Beginn der Schwangerschaft sowie die Zunahme von Adipositas und Typ-2-Diabetes in der Schwangerschaft.

Ein präkonzeptionell bestehender Diabetes mellitus wird angenommen, wenn vor der 20. Schwangerschaftswoche die Kriterien für einen manifesten Diabetes erfüllt sind: Nüchternblutzucker ≥ 126 mg/dl, Gelegenheitsmessung oder 2‑Stunden-Wert im oGTT ≥ 200 mg/dl, HbA_1c_ ≥ 6,5 %. Patientinnen, bei denen sich während der Schwangerschaft ein Typ-1-Diabetes manifestiert oder bei denen ein anderer Diabetestyp erst in der Schwangerschaft diagnostiziert wird („diabetes in pregnancy“ [DIP]), sollen wie Frauen mit bekanntem präkonzeptionellem Diabetes überwacht und behandelt werden (Abb. 1).

**Abb. 1:**
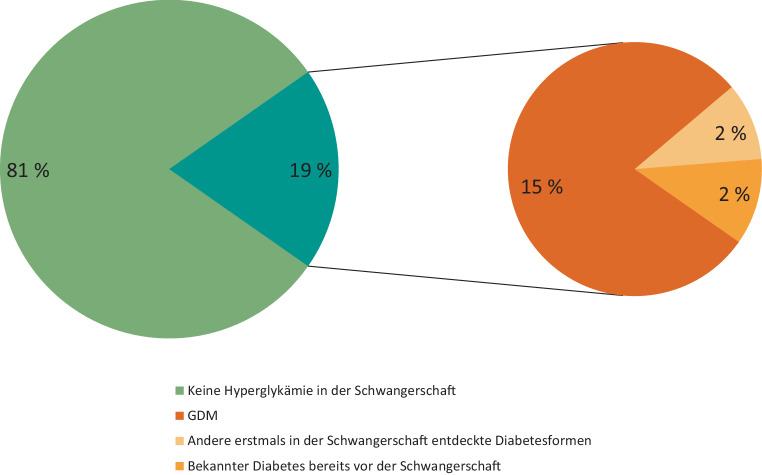
Anteil an Hyperglykämie in der Schwangerschaft weltweit 2024. Die altersstandardisierte Prävalenz beträgt 19,7 % [122]

Auch Frauen mit Gestationsdiabetes (GDM) – einer erstmals in der Schwangerschaft aufgetretenen Glukosetoleranzstörung – weisen im Vergleich zu Frauen mit normaler Glukosetoleranz ein erhöhtes Risiko für perinatale Komplikationen, operative Entbindungen sowie langfristig für die Entwicklung eines Typ-2-Diabetes (T2DM) und kardiovaskuläre Erkrankungen auf [1, 2].

Eine möglichst normoglykämische Stoffwechsellage ist während der gesamten Gravidität und unter der Geburt essenziell. Studien zeigen, dass auch bei GDM Frauen mit guter Stoffwechselkontrolle signifikant bessere Schwangerschaftsergebnisse erzielen als unbehandelte Frauen [3, 4]. Antenatale Lifestyle-Interventionen, insbesondere strukturierte Ernährungsberatung und Bewegungsprogramme sowie eine Gewichtsoptimierung, tragen zur Verbesserung der Outcomes bei [5].

Eine Betreuung durch ein interdisziplinäres Team mit Erfahrung in der Behandlung schwangerer Patientinnen mit Diabetes sowie die Entbindung in einem Zentrum mit neonataler Intensivstation werden empfohlen, um das Risiko für Mutter und Kind zu minimieren.

## Prävention des GDM und Schwangerschaftsplanung bei erhöhtem Risiko

### GDM-Prävention

Die Auswirkungen von Maßnahmen zur Prävention eines Gestationsdiabetes (GDM) wurde in zahlreichen Studien insbesondere bei Risikogruppen wie adipösen Frauen untersucht. In großen randomisierten Studien zeigte sich, dass Lebensstilinterventionen bei adipösen Schwangeren weder das Risiko für GDM noch fetale Outcomes wie „large for gestational age“ (LGA) signifikant verbessern konnten [6, 7].

Die DALI-Studie untersuchte die Effektivität einer gesunden Ernährung und körperlicher Aktivität hinsichtlich maternaler und neonataler Risiken. Zwar kam es in der kombinierten Interventionsgruppe zu einer signifikanten Reduktion der Gewichtszunahme, dies beeinflusste jedoch nicht die maternalen oder neonatalen Outcomes [6, 7]. Metaanalysen können aber durchaus positive Effekte mit Senkung des Risikos für GDM von Ernährungs‑, Bewegungs- oder kombinierten Lebensstilinterventionen zeigen (Risikoreduktion jeweils 25 %, 31 % und 18 %) [8]. Insbesondere kombinierte Ernährungs- und Bewegungsprogramme bei Frauen mit GDM-Risikofaktoren führten zu einer deutlichen Risikoreduktion [9].

Eine Supplementation mit Vitamin D führte im dritten Trimenon zwar zu einer Senkung der Nüchternglukose, hatte jedoch keine klinisch relevante Auswirkung auf GDM-Inzidenz oder LGA-Risiko [10]. Auch Lipidparameter blieben unbeeinflusst [11]. Die Gabe von Probiotika und Myo-Inositol konnte in Metaanalysen das Risiko für GDM zwischen 12 % (Probiotika) und 61 % (Myo-Inositol) senken, v. a. bei adipösen Frauen oder solchen mit positiver Familienanamnese [8]. Eine Cochrane Analyse bestätigte positive Effekte einer Myo-Inositol-Therapie zur Reduktion von GDM, allerdings bei sehr niedrigem Evidenzlevel [12]. Bei adipösen Schwangeren zeigte Metformin in einer randomisiert kontrollierten Studie keine präventive Wirkung auf das GDM-Risiko oder auf geburtshilfliche Outcomes [6]. Eine Metanalyse mit 13 Studien konnte aber eine Reduktion des GDM-Risikos um 36 % zeigen [8].

Die bisherigen Daten unterstreichen, dass präventive Maßnahmen bzw. ein gesunder Lebensstil idealerweise bereits vor Eintritt der Schwangerschaft bzw. im ersten Trimenon begonnen werden sollten [5, 6].

Bestimmte Lebensstilfaktoren vor der Schwangerschaft – wie gesunde Ernährung (Zucker-reduziert bzw. hohe Ernährungsqualität) und hohe körperliche Aktivität – können das Risiko für Gestationsdiabetes zwischen 14 und 29 % senken [13]. Die Gabe von Myo-Inositol in der Dosierung von 2‑mal 2 g pro Tag kann erwogen werden, um das Auftreten von GDM zu verringern.

### Schwangerschaftsplanung bei vorbestehendem Diabetes, prä- und perikonzeptionelle Betreuung

Eine Schwangerschaft bei Frauen mit Diabetes betrifft zunehmend auch Frauen mit Typ-2-Diabetes mellitus (T2DM) neben dem weiterhin in dieser Population häufigeren Typ-1-Diabetes (T1DM). Adipositas, höheres maternales Alter und arterielle Hypertonie verschlechtern das Outcome zusätzlich [14–16]. Der BMI von Frauen mit T1DM ist in den letzten Jahren ebenfalls signifikant angestiegen [16].

Höherer BMI, längere Diabetesdauer und Hypertonie zu Schwangerschaftsbeginn sind mit schlechteren Schwangerschaftsergebnissen assoziiert [17]. Besonders vulnerabel sind Frauen mit Migrationshintergrund oder niedrigem sozioökonomischem Status, bei denen eine adäquate Betreuung vor der Schwangerschaft oft fehlt [18].

Trotz vergleichbarer Raten für kongenitale Fehlbildungen und Totgeburten bei T1DM und T2DM treten Frühgeburten und LGA-Neugeborene häufiger bei T1DM auf; bei T2DM ist jedoch die neonatale Mortalitätsrate erhöht [18]. Ein HbA_1c_ ≥ 6,5 % im 3. Trimester, T2DM und niedriger sozioökonomischer Status erhöhen unabhängig das Risiko für perinatalen Tod [19].

#### Wichtige Maßnahmen bei Frauen im reproduktiven Alter und Diabetes

**Abb. 2:**
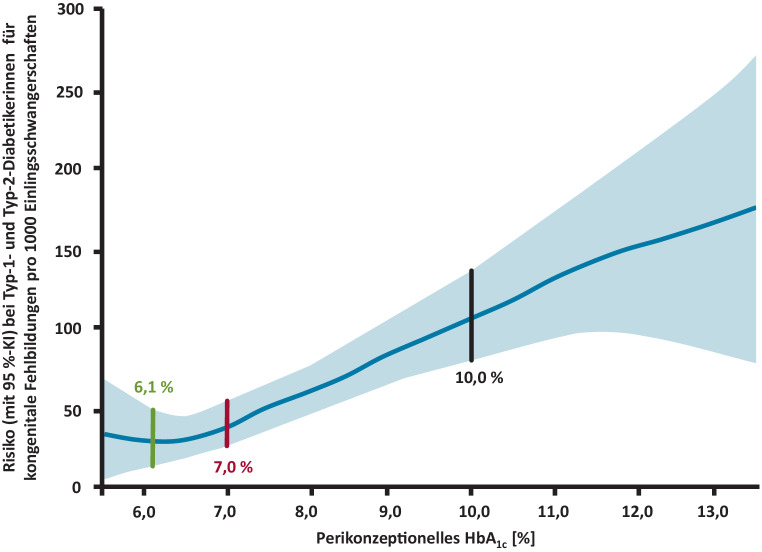
Zusammenhang von perikonzeptionellem HbA_1c_ bei Frauen mit Diabetes und dem Risiko einer kongenitalen Fehlbildung (nach [22]). Das National Institute for Health and Clinical Excellence (UK) empfiehlt einen HbA_1c_ unter 6,1 %, die American Diabetes Association (USA) unter 7,0 % bei Frauen mit Diabetes, die eine Schwangerschaft planen. Bei einem HbA_1c_ über 10 % regt das National Institute for Health and Clinical Excellence an, den Frauen mit Diabetes dringend von einer Schwangerschaft abzuraten. *Linie* Risiko, *blaue Schattierung* 95 %-Konfidenzintervall

- Schwangerschaft soll geplant sein, sollte kein Kinderwunsch bestehen, werden Verhütungsmaßnahmen empfohlen
- Optimale Glukoseeinstellung (HbA_1c_ < 6,5 %) bereits bei Kinderwunsch [20, 21]. Normoglykämie anstreben, wenn ohne Hypoglykämien erreichbar
- Ab HbA_1c_ > 8 % ist das Risiko für Fehlbildungen und perinatale Mortalität (6-fach bei HbA_1C_ über 9 %) stark erhöht (Abb. 2; [22])
- Risiko steigt linear: + 30 % Fehlbildungen pro 1 % HbA_1c_ über 6,3 % [22]

Menschen mit Diabetes haben in der Regel die gleichen Verhütungsoptionen wie Personen ohne Diabetes [20]. Frauen nach GDM sollen reine Gestagenpräparate insbesondere in der Stillzeit vermeiden, da sich dadurch das Risiko für die Manifestation eines T2DM erhöhen könnte [23]. Außer auf eine Glukosestoffwechselstörung soll auch auf weitere kardiovaskuläre Risikoparameter wie Dyslipidämie und Hypertonie untersucht werden, da Frauen nach GDM ein höheres kardiovaskuläres Risiko aufweisen [24, 25]. Bei bestehenden Gefäßkomplikationen kann die Art der Verhütung angepasst werden. Lang wirksame, reversible Methoden (z. B. Spirale, Implantat) sind oft besonders geeignet. Das Risiko einer ungewollten Schwangerschaft überwiegt meist das Risiko der Verhütung selbst.

Moderne Technologien wie die kontinuierliche Glukosemessung (CGM) und Therapien mit Insulinpumpen bzw. AID-Systemen ermöglichen eine engmaschige Stoffwechselkontrolle und sollten bei Diabetes bereits präkonzeptionell etabliert sein [26]. Die Kombination aus CGM und Insulinpumpen bzw. AID-Systemen kann die glykämische Kontrolle verbessern, die Zeit im Zielbereich erhöhen und das Risiko für LGA-Geburten sowie mütterliche und neonatale Hypoglykämie senken [26]. Wichtig ist die korrekte Einstellung des Zielbereichs bei kontinuierlicher Glukosemessung (Tab. 1). Dieser liegt zwischen 63 und 140 mg/dl bzw. zwischen 63 und 130 mg/dl bei auffälligen Biometriedaten bei schwangeren Frauen und sollte > 70 % („time in range“) im Zielbereich bei schwangeren Frauen mit T1DM und noch viel höher bei T2DM und GDM sein [27]. Die Zeit unter dem Zielbereich („time below range“) sollte so gering wie möglich sein, aber zumindest < 4 % [27].

**Tab. 1:** Glukosezielwerte bei kapillärer Messung und Sensormessung [20, 30, 62]

*Zeitpunkt*	*Kapilläres Vollblut (mg/dl)*
Nüchtern (und präprandial)	65–95
1 h postprandial	< 140
2 h postprandial	< 120
Vor dem Schlafengehen, ca. 22:00–23:00 Uhr	90–120
Nachts in der Zeit von 2:00–4:00 Uhr	> 65
**CGMS-Glukosemessung**	
*Ziel (%)*	*Zielbereich Sensormessung (mg/dl)*
TIR > 70 %	63–140
Level 1 TBR < 4 %	< 63
Level 2 TBR < 1 %	< 54
TAR < 25 %	> 140

*CGMS* kontinuierliche Glukosemessung („Sensormessung“), *TIR* Zeit im Zielbereich, *TBR* Zeit unter Zielbereich, *TAR* Zeit über Zielbereich

#### Allgemeine Maßnahmen (einschließlich post GDM)

Bei bestehendem Kinderwunsch müssen alle laufenden bzw. geplanten Medikamente überprüft werden. Blutdrucksenker wie ACE-Hemmer, AT_1_-Rezeptorblocker und Statine sind potenziell teratogen und sollten vor einer Konzeption durch in der Schwangerschaft zugelassene Alternativen ersetzt werden. Auch orale Antidiabetika sollten kritisch evaluiert und bei Bedarf auf Insulin umgestellt werden [20].

Frauen mit T2DM und Kinderwunsch sollten bereits präkonzeptionell auf eine Insulintherapie umgestellt werden. Eine entsprechende Schulung der Patientinnen zur Selbstanpassung der Insulindosis und Aufklärung über mögliche Risiken sowie die zu erwartenden Stoffwechselveränderungen in der Schwangerschaft sollten durch die betreuenden Ärzt:innen bei Schwangerschaftsplanung/Kinderwunsch erfolgen.

Im Falle einer ungeplanten Gravidität bei T2DM unter Einnahme oraler Antidiabetika (OAD) gibt es bisher keine Evidenz für ein erhöhtes Missbildungsrisiko durch OAD, jedoch ist zu bedenken, dass Metformin plazentagängig ist und bezüglich Langzeitfolgen bei den Nachkommen widersprüchliche Erkenntnisse vorliegen [20, 28]. In der MOMPOD-Studie [29] führte die Kombination von Metformin und Insulin bei Schwangeren mit vorbestehendem T2DM oder früh diagnostiziertem Schwangerschaftsdiabetes zu keiner signifikanten Reduktion unerwünschter neonataler Ergebnisse im Vergleich zur alleinigen Insulintherapie. Allerdings zeigte sich unter Metformin eine geringere Wahrscheinlichkeit, ein überdurchschnittlich großes Neugeborenes (LGA) zu gebären [29]. Bei sehr insulinresistenten und stark übergewichtigen Frauen mit T2DM kann damit eine zusätzliche Therapie mit Metformin überlegt werden, um den Stoffwechsel zu verbessern und die Insulinresistenz zu mildern [30]. Für den Einsatz anderer OADs kann keine Empfehlung abgegeben werden.

GLP-1-Rezeptoragonisten, die bei Frauen mit T2DM oder Frauen mit Adipositas (inklusive Frauen post GDM) verwendet werden, sollen vor der Konzeption abgesetzt werden und die antihyperglykämische Therapie angepasst werden, da teratogene Effekte im Tiermodell beschrieben wurden und die Anwendung in der Schwangerschaft nicht empfohlen wird. Präliminäre Anwendungsdaten zu Schwangerschaftsbeginn zeigen aber keinen Hinweis auf ein erhöhtes Risiko für Fehlbildungen [31]; Andererseits war eine Therapie mit GLP-1-Rezeptoragonisten innerhalb von 24 Monaten vor einer Schwangerschaft mit besseren Schwangerschafts-Outcomes und weniger GDM-Diagnosen verbunden [32]. Es ist jedenfalls ein erhöhtes Risiko für rasche Gewichtszunahme und Verschlechterung der Glykämie nach Absetzen von Inkretinmimetika zu beachten, was ebenso mit einem höheren Risiko für Schwangerschaftskomplikationen verbunden ist [33].

Vor dem Absetzen der Kontrazeption sollte bei Diabetes eine umfassende internistische Abklärung erfolgen. Dazu zählen die Kontrolle der Retinopathie (Augenhintergrund), Nephropathie (Nierenfunktion), kardiovaskuläre Risikoevaluierung (inklusive EKG oder kardiologischer Kontrolle bei Vorerkrankungen) sowie die Bestimmung von TSH, Lipiden und Mikroalbuminurie (Albumin/Kreatinin-Ratio im Harn) [20, 34]. Ziel ist es, vor Schwangerschaftsbeginn mögliche Risiken frühzeitig zu erkennen und zu minimieren.

Die Einnahme von Folsäure (400–800 µg/Tag) bereits bei Kinderwunsch bis einschließlich der 12. Schwangerschaftswoche ist obligat. Bei Adipositas oder T2DM werden bis zur 12. Schwangerschaftswoche sogar höhere Dosen (5 mg täglich) empfohlen [35, 36].

## Risikoevaluierung und Diagnose des GDM und Diabetes in der Schwangerschaft

### Frühschwangerschaft

**Abb. 3:**
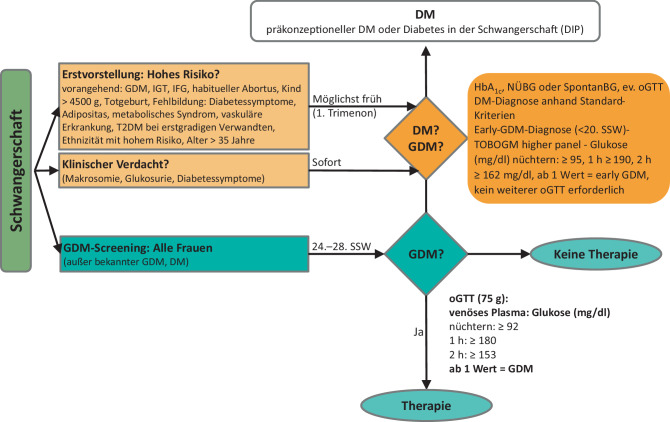
Flussdiagramm Gestationsdiabetes (*GDM*) – Risikoevaluierung und Diagnose. *DM* Diabetes mellitus, *GDM* Gestationsdiabetes, *IGT* gestörte Glukosetoleranz, *IFG* gestörte Nüchternglukose, *SSW* Schwangerschaftswoche, *oGTT* oraler Glukosetoleranztest, *NüBG* Nüchternglukose, *HbA*_*1c*_ Langzeitzucker, *BG* Blutglukose

Bei Erstvorstellung sollen schwangere Frauen mit erhöhtem Risiko für Hyperglykämie in der Schwangerschaft auf das Vorliegen einer Glukosestoffwechselstörung untersucht werden (Abb. 3):

Hohes Risiko für GDM bzw. Risiko für vorbestehende, unerkannte Stoffwechselstörung (Prädiabetes oder Diabetes mellitus) besteht bei:

- GDM in einer früheren Schwangerschaft,
- Prädiabetes in der Anamnese (gestörte Glukosetoleranz und/oder Nüchternglukose > 100 mg/dl),
- kongenitale fetale Fehlbildung in einer früheren Schwangerschaft,
- Geburt eines Kindes > 4500 g,
- intrauteriner Fruchttod (IUFT),
- habitueller Abortus (> 3 Fehlgeburten hintereinander),
- Diabetessymptome,
- Adipositas (BMI ≥ 30 kg/m^2^),
- Alter über 35 Jahre,
- metabolisches Syndrom,
- vaskuläre Erkrankung (koronare Herzkrankheit [KHK], zerebrovaskuläre Verschlusskrankheit [cAVK], periphere arterielle Verschlusskrankheit [pAVK]),
- Familienanamnese von T2DM bei erstgradigen Verwandten,
- Ethnizität (z. B. arabisch, S‑ und SO-asiatisch, lateinamerikanisch).

Die Untersuchung kann durch eine Nüchternglukosemessung, eine HbA_1c_-Bestimmung und/oder die Durchführung eines oGTT erfolgen. Bei Nüchternglukose ≥ 126 mg/dl, Spontanglukose/2 h Glukosewert im oGTT ≥ 200 mg/dl oder HbA_1c_ ≥ 6,5 % liegt ein manifester Diabetes vor.

Bei Auftreten von diabetesspezifischen Symptomen oder klinischen Auffälligkeiten (Polydipsie, Polyurie, Glukosurie; Makrosomie) ist ein Glykämietest – auch bei unauffälligem Vorbefund und unabhängig von der Schwangerschaftswoche – unmittelbar durchzuführen.

Die TOBOGM-Studie [37] untersuchte die Auswirkungen einer frühzeitigen Behandlung des Gestationsdiabetes mellitus (GDM) vor der 20. Schwangerschaftswoche. Eine frühe Diagnose eines Gestationsdiabetes, ein sog. early GDM, ist mit einem erhöhten Risiko für Geburtskomplikationen assoziiert [37]. Dabei wurden 2 unterschiedliche Diagnosekriterien für GDM verwendet: ein niedrigerer Schwellenwert („lower panel“) basierend auf den IADPSG-Kriterien und ein höherer Schwellenwert („higher panel“).

Angelehnt an die Resultate der TOBOGM-Studie [37] empfehlen wir sowie andere diabetologische Fachgesellschaften [38] bei Frauen mit hohem Risiko wie Adipositas und/oder einer Vorgeschichte von GDM, bei denen bereits vor der 20. Schwangerschaftswoche ein 75 g-oGTT durchgeführt wurde und bei welchen Blutzuckerwerte über den unten dargestellten Referenzwerten („higher panel“) im frühen oGTT festgestellt wurden, einen GDM zu diagnostizieren (= early GDM) und mit einer Behandlung zu beginnen.

#### Grenzwerte des „higher panel“ nach TOBOGM

- Nüchternblutzucker: ≥ 95 mg/dl (5,3 mmol/l)
- 1‑h-Wert: ≥ 190 mg/dl (10,6 mmol/l)
- 2‑h-Wert: ≥ 162 mg/dl (9,0 mmol/l)

#### Warum wird eine Behandlung ab den Referenzwerten des „lower panel“ nach TOBOGM nicht mehr empfohlen?

Die Anwendung der niedrigeren IADPSG-Grenzwerte („lower panel“) in der frühen Schwangerschaft führt zu einer höheren Anzahl diagnostizierter GDM-Fälle. Allerdings zeigte die TOBOGM-Studie, dass die Behandlung von Frauen, die nur nach diesen niedrigeren Kriterien diagnostiziert wurden, nicht zu einer signifikanten Reduktion neonataler Komplikationen führte [37]. In dieser Subgruppe stieg das Risiko für SGA an.

#### Schwangerschaftswoche 24–28

Alle Schwangeren erhalten im Rahmen der Mutter-Kind-Pass-Untersuchungen zwischen der 24. und 28. Schwangerschaftswoche einen 2 h-75 g-oGTT [20, 30, 39]. Die internationale Klassifikation (Tab. 2; [39–41]) beruht auf evidenzbasierten (= HAPO-Studie) Blutzuckergrenzwerten [42, 43]. Ab einem pathologischen Wert ist ein GDM diagnostiziert.

**Tab. 2:** GDM-Diagnosekriterien nach 75 g oGTT (nach WHO, IADPSG und ADA-Empfehlung [39–41]).

Zeitpunkt	Venöses Plasma (mg/dl)
Nüchtern	≥ 92 (5,1 mmol/l)
1 h	≥ 180 (10,0 mmol/l)
2 h	≥ 153 (8,5 mmol/l)

*ADA* American Diabetes Association, *GDM* Gestationsdiabetes, *IADPSG* International Association of the Diabetes and Pregnancy Study Groups, *WHO* World Health Organization

Bei Frauen mit bereits diagnostiziertem GDM oder Diabetes bzw. wenn der unmittelbar gemessene Nüchternglukosewert (venöse Plasmaglukose) ≥ 92 mg/dl ist, sollte der oGTT *nicht *durchgeführt werden. Ausgenommen von der Durchführung eines oGTT sind weiters Frauen nach bariatrischer/metabolischer Chirurgie, da das Risiko einer postprandialen Hypoglykämie (Dumping-Phänomen) nach der Ingestion der Glukoselösung besonders hoch ist [35]. Nach bariatrischer Operation werden daher regelmäßige Blutzuckerselbstkontrollen (2-wöchiges 4‑Punkt-Blutzuckertagesprofil, Glukosezielwerte, Tab. 1) zur Diagnose eines GDM herangezogen. Ebenso ist die Verwendung eines kontinuierlichen Glukosemesssystems (CGMS) in diesem Fall denkbar [35].

In einer multizentrischen österreichischen Studie waren ein GDM in einer früheren Schwangerschaft, das Auftreten einer Glukosurie, Übergewicht (präkonzeptioneller BMI > 27 kg/m^2^), ein Alter über 30 Jahre und der Verdacht auf Makrosomie im Ultraschall die besten unabhängigen Prädiktoren für einen GDM [44], wobei das Risiko bei vorangegangenem GDM fast 3‑fach, ansonsten ungefähr 2‑fach erhöht war. Eine multinationale europäische Studie zeigte, dass fast jede 4. adipöse Frau bereits vor der 20. Schwangerschaftswoche erhöhte Blutzuckerwerte im Sinne eines GDMs nach IADPSG/WHO 2013-Kriterien und Parameter des metabolischen Syndroms aufwies [39–41].

Durch die Einführung des oGTTs im Mutter-Kind-Pass konnte in Österreich eine Reduktion der IUFT-Fälle bei schwangeren Frauen mit einem erhöhten Risiko für GDM erreicht werden [42, 43].

### Methodik: diagnostischer 75 g oraler Glukosetoleranztest (oGTT)

Der Test soll bei allen Frauen mit bisher unauffälligen oder unbekannten Blutglukosewerten in der Schwangerschaft morgens nach mindestens 8‑stündiger Nahrungskarenz durchgeführt werden. Eine Änderung der bisherigen Ernährung, eine Reduktion der Kohlenhydrate oder Diäten vor dem Test sollten vermieden werden. Ebenso sollten vor dem Test keine außergewöhnlichen körperlichen Belastungen erbracht werden. Der Testbeginn sollte zwischen 6.00 h und 9.00 Uhr erfolgen, da die Glukosetoleranz tageszeitlichen Änderungen unterliegt. Die Schwangere soll die Glukoselösung (75 g Glukose in 300 ml Wasser) innerhalb von 5 min trinken, während des Testes sitzen (liegende Position vermeiden, keine unnötige körperliche Aktivität) und nicht rauchen.

Zur GDM-Diagnostik sollen Blutglukosewerte ausschließlich mit einer qualitätsgesicherten Methode in venösem Plasma direkt oder in venösem Vollblut (mit einem Faktor von 1,11 [+ 11 %] in venöse Plasmawerte umgerechnet) gemessen werden. Um möglichst exakte oGTT-Resultate zu erhalten, ist es erforderlich gewisse Standards zu berücksichtigen [30]: Diese sind wie folgt (abgeleitet nach [30]):

- Messungen aus venösem Plasma und nicht aus Kapillarblut
- Messung in einem zertifizierten Labor nach zertifizierten Methoden, um präanalytische Fehler zu minimieren
- Am Testtag ist vor dem oGTT eine Einnahme kontrainsulinärer Medikamente (z. B. Thyroxin, Progesteron, Glukokortikoide, Sympathomimetika) zu vermeiden
- Nach Einleitung der fetalen Lungenreife mittels Glukokortikoiden sollte man bis zur Testdurchführung mindestens 5 Tage zuwarten
- Bei Fieber, akuten Erkrankungen oder verordneter Bettruhe ist der Test bis zur vollständigen Genesung zu verschieben
- Bei Hyperemesis gravidarum oder stärkerer Schwangerschaftsübelkeit ist der Test um einige Tage zu verschieben

### Glukosemanagement bei Hyperglykämie in der Schwangerschaft

Während der Schwangerschaft soll versucht werden, individualisiert die bestmögliche Stoffwechsellage unter Berücksichtigung der Hypoglykämiewahrnehmung und -häufigkeit, der individuellen Fähigkeiten sowie der Lebensumstände mit normoglykämischen Blutzuckerwerten (Tab. 1) zu erreichen. Lebensstilmaßnahmen mit regelmäßiger körperlicher Aktivität und Ernährungsoptimierung sind auch bei Diabetes in der Schwangerschaft zu empfehlen, und eine diätologische Beratung soll den schwangeren Frauen mit Diabetes angeboten werden (Kapitel Ernährung bei Diabetes in der Schwangerschaft).

Generell ist bei Frauen mit präkonzeptionellem Diabetes mellitus eine Insulintherapie in der Schwangerschaft notwendig [20]. Zu Beginn der Schwangerschaft ist die Hypoglykämierate relativ groß und die Insulindosis vorsichtig anzupassen. Insbesondere bei Frauen mit T1DM ist das Risiko für schwere Hypoglykämien in der Frühschwangerschaft 3‑ bis 5‑fach höher als vor der Schwangerschaft [45]. In der CONCEPTT-Studie zeigten 30 % der Schwangeren mit T1DM eine verminderte Hypoglykämiewahrnehmung, was mit mehr hypoglykämischen Episoden, größerer Glukosevariabilität und Hypoglykämieangst/Diabetes-Distress trotz CGM verbunden war [46].

Generell gilt, dass im Lauf der Gravidität (üblicherweise beginnend mit der 20. Schwangerschaftswoche) die Insulintagesdosis auf 50–100 %, bei adipösen Frauen mit T2DM oft noch höher angehoben werden muss, um die zunehmende Insulinresistenz zu kompensieren und die empfohlenen Blutzuckerzielwerte in der Schwangerschaft zu erreichen (Tab. 1).

#### Blutglukosemanagement

Schulung in Blutzuckerselbstmessung. Dokumentation der Glukoseprofile: mindestens 4 Messungen täglich (nüchtern, 1 h postprandial) bei GDM, bei manifestem Diabetes sind meist deutlich mehr Messungen notwendig inklusive einer BG-Messung vor dem Schlafengehen, weswegen zu einem CGM geraten wird. Bei Beginn einer Insulintherapie sollte die Patientin über die Symptome und das Risiko von Hypoglykämien sowie über das richtige Verhalten in dieser Situation geschult werden. Eine fehlende Adhärenz zu regelmäßigen selbstständigen Blutzuckermessungen steht bei Frauen mit GDM mit einem erhöhten Präeklampsierisiko in Zusammenhang, insbesondere bei niedrigem sozioökonomischem Status, nichteuropäischer Herkunft und Diabetes in der Familienanamnese [47]. Hingegen konnten bei guter Adhärenz mit guter Blutzuckereinstellung keine Unterschiede zwischen täglich 4 Messungen und Messungen jeden zweiten Tag in mütterlichen und kindlichen Outcomes festgestellt werden [48]. Eine Reduktion der Blutzuckermessungen (Messung alle 2 Tage) kann bei GDM und guter Blutzuckereinstellung und fortgeschrittener Schwangerschaft überlegt werden. Andere Fachgesellschafen und wir empfehlen bei GDM bei Blutglukosezielwerten im Zielbereich über einen Zeitraum von 2 Wochen eine Reduktion auf eine 1‑mal tägliche Messung im Rotationsverfahren (Stufenprotokoll mit abwechselnden Messungen nüchtern, 1 h postprandial morgens, mittags, abends) [30]. Zusätzliche gezielte Messungen nach Erfordernis sind möglich.

Bei kontinuierlicher Glukosemessung sind folgende Blutzuckerwerte anzustreben: Zielbereich 63–140 mg/dl bzw. 63–130 mg/dl bei auffälliger Biometrie, Zeit im Zielbereich („time in range“) > 70 % (noch höher bei GDM und T2DM), Zeit unter Zielbereich < 4 %, Zeit über Zielbereich < 25 % [20]. CGM-Werte außerhalb des Zielbereichs, v. a. < 63 mg/dl, sollen mittels kapillärer Messungen kontrolliert werden, bevor sie zu einer Therapieänderung führen [20].

Werden bei GDM die Grenzwerte zu einem Messzeitpunkt zu 50 % überschritten (d. h. z. B. 6 von 12 Messungen überschreiten die in Tab. 1 dargestellten Grenzwerte), ist eine individuell anzupassende Insulintherapie zu beginnen. Liegen Nüchternglukosewerte über 110 mg/dl vor, ist ein sofortiger Therapiebeginn mit Insulin erforderlich [30]. Der HbA_1c_-Wert ist für die Diagnose eines GDM ungeeignet, da er durch schwangerschaftstypische Faktoren wie Anämie und gesteigerte Erythropoese beeinflusst werden kann. Zudem erfasst der HbA_1c_ die für GDM typischen postprandialen Blutzuckerspitzen nicht, die jedoch eine zentrale Rolle für die fetale Entwicklung spielen. Der HbA_1c_ kann aber zur Verlaufskontrolle der Metabolik herangezogen werden und soll jedenfalls im Referenzbereich für Gesunde liegen (HbA_1c_ < 6,0 %) [20].

Die mütterlichen Blutzuckerprofile sollen auch während der Geburt im Zielbereich liegen (90–140 mg/dl bzw. strengere Zielwerte bei auffälliger Biometrie) [49], um neonatale Hypoglykämien und Anpassungsstörungen zu vermindern. Bei mit Ernährungsberatung gut eingestelltem GDM ist keine routinemäßige Glukosekontrolle während der Geburt erforderlich. Bei insulinpflichtigem GDM sollten Blutzuckerkontrollen alle 2 h erfolgen [49]. In der Regel besteht kein Insulinbedarf unter der Geburt. Postpartal wird die Insulintherapie beendet, ein 4‑Punkte-Tagesprofil sollte für einige Tage weitergeführt werden und bei wiederholt erhöhten Werten (nüchtern > 126 mg/dl, postprandial > 200 mg/dl) diabetologisch vorgestellt werden [49]. Bei T1DM sollten engmaschigere Glukosekontrollen bei der Geburt durchgeführt werden und, wenn erforderlich, schnell wirksames Insulin zur Korrektur verabreicht werden.

Zur weiteren Individualisierung der Therapie dient die fetale Biometrie, die zur Entscheidung, ob eine medikamentöse Therapie begonnen, intensiviert oder gelockert werden muss, herangezogen werden soll [30]. Der fetale Abdomenumfang korreliert mit dem fetalen Insulinspiegel. Liegt eine fetale asymmetrische Wachstumssteigerung vor und liegt die abdominelle Zirkumferenz über der 75. Perzentile des Gestationsalters, sind strengere Therapieziele anzustreben. Hierbei sollen Nüchternglukosewerte bei kapillärer Messung < 90 mg/dl und 1 h postprandial < 130 mg/dl erreicht werden. Bei fetaler Wachstumsretardierung sind dementsprechend auch individuell angepasste, höhere mütterliche Glukosegrenzwerte zulässig bzw. ist der Beginn einer Insulintherapie zurückhaltend zu wählen [50].

Regelmäßige Kontrollen sollen individuell, den Bedürfnissen der schwangeren Frauen entsprechend, im Abstand von wenigen Tagen bis 3 Wochen erfolgen. Dabei ist anhand der Blutglukoseprofile eine Therapieanpassung (Insulindosis) je nach Erfordernis durchzuführen. Der Blutdruck und die Gewichtszunahme sollten kontrolliert und ein Harnbefund sollte erhoben werden. Auch ergänzende telemedizinische Visiten können die Betreuungsqualität erhöhen und die Therapiekontrolle vereinfachen. Telemedizinische Visiten bei GDM konnten in einer Metaanalyse das Risiko für GDM-assoziierte Geburts- und Schwangerschaftskomplikationen im Vergleich zur Standardbetreuung reduzieren [20, 51].

### Therapie

#### Ernährung bei Diabetes in der Schwangerschaft

**Abb. 4:**
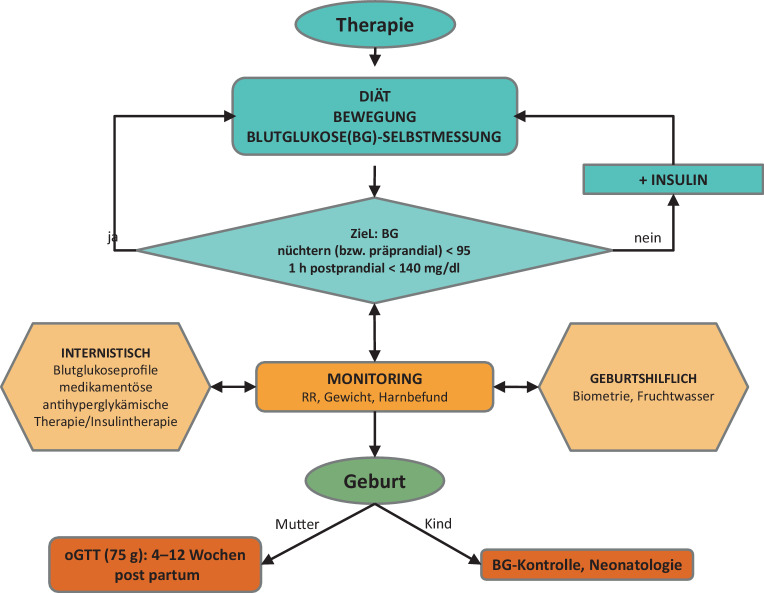
Flussdiagramm Gestationsdiabetes (*GDM*) – Behandlung. *BG* Blutglukose, *RR* Blutdruck, *oGTT* oraler Glukosetoleranztest

Eine strukturierte, individuell angepasste Ernährungstherapie ist bei Diabetes in der Schwangerschaft essenziell [20, 30]. Durch eine ausgewogene Nährstoffzufuhr, passende Kohlenhydratverteilung, regelmäßige Mahlzeiten und Gewichtskontrolle können sowohl die mütterliche Blutzuckerkontrolle als auch die fetale Entwicklung positiv beeinflusst werden – oft sogar ohne Insulintherapie (Abb. 4).

Die erste therapeutische Maßnahme bei vorbestehendem Diabetes oder Gestationsdiabetes (GDM) in der Schwangerschaft ist eine individuelle Ernährungsevaluation und -beratung, idealerweise durch eine:n erfahrene:n Diätolog:in [20, 30]. Diese sollte sich an den Prinzipien einer ausgewogenen, nährstoffreichen Ernährung für Schwangere orientieren. Als Basis dafür dienen die entsprechenden nationalen Referenzwertempfehlungen für Nahrungszufuhr in der Schwangerschaft (D-A-CH-Referenzwerte, www.oege.at).

Die Ernährungstherapie verfolgt folgende Ziele:

- normnahe, schwangerschaftsspezifische Blutzuckerwerte ohne Hypoglykämien oder Ketose,
- eine angemessene, BMI-abhängige Gewichtszunahme,
- ein gesundes fetales Wachstum.

#### Nährstoffverteilung und -qualität

Die Ernährung sollte dem erhöhten Nährstoffbedarf der Schwangerschaft entsprechen und kalorisch ausreichend, aber nicht überversorgend sein. Empfohlene Makronährstoffverteilung:

- Kohlenhydrate: 40–50 % der Gesamtenergie (nicht unter 40 % bzw. 175 g/Tag),
- Eiweiß: ca. 20 %,
- Fett: 30–35 % (mit Fokus auf ungesättigte Fettsäuren, z. B. n‑3-Fettsäuren aus Fisch, Nüssen, Samen).

Bevorzugt werden hochwertige Kohlenhydrate mit niedrigem glykämischem Index und hohem Ballaststoffanteil, wie sie in Vollkornprodukten, Gemüse, Hülsenfrüchten und Obst vorkommen. Einfache Zucker sowie stark verarbeitete Lebensmittel, zuckerhaltige Getränke und fettreiches rotes Fleisch sollten reduziert werden. Für eine DASH- oder mediterrane Ernährung werden sowohl präventive als auch Outcome-orientiert positive Daten bezüglich GDM berichtet [52]. Die minimale Aufnahme liegt zwischen 1500 und 2000 kcal/Tag [52]. Bei einer Low-Carb-Ernährung mit nur 35–40 % Kalorien aus Kohlenhydraten wird eine ausgleichende Protein- und Fettzufuhr vorwiegend pflanzlichen Ursprungs empfohlen.

Werden Kohlenhydrate durch Fett ersetzt, kann dies die Lipolyse verstärken, der Spiegel freier Fettsäuren erhöht und dadurch die Insulinresistenz der Mutter verschlechtert werden [53]. In einer Sekundäranalyse einer GDM-Präventionsstudie konnte in der Ernährungsinterventionsgruppe mit reduzierter Kohlenhydratzufuhr zwar eine niedrigere Gewichtszunahme in der Schwangerschaft, aber auch ein Zusammenhang mit vermutlich Lipolyse-induzierten höheren Nüchternglukosewerten, freien Fettsäuren und Ketonkörpern beobachtet werden [54]. Auch im Nabelschnurblut konnten erhöhte freie Fettsäuren festgestellt werden, wobei Langzeitbeobachtungen leider fehlen.

#### Mahlzeitenstruktur

Zur besseren Blutzuckerkontrolle wird empfohlen (nach [30]):

- Verteilung der Kohlenhydrate auf 3 Haupt- und 2 bis 3 kleine Zwischenmahlzeiten pro Tag,
- Kohlenhydratreduktion zum Frühstück, da hier der stärkste postprandiale Glukoseanstieg auftritt,
- Spätmahlzeit mit ca. 1 KE (ca. 10–12 g KH), um nächtlicher Ketonkörperbildung vorzubeugen.

Auf eine ausreichende Versorgung mit Vitaminen und Mineralstoffen (z. B. Folsäure, Eisen, Jod, Vitamin D, Kalzium, Magnesium) ist zu achten. Hier gelten die nationalen Referenzwertempfehlungen für Nahrungszufuhr in der Schwangerschaft (D-A-CH-Referenzwerte, www.oege.at) Die täglich empfohlene Proteinzufuhr in der Schwangerschaft entspricht der einer gesunden Schwangerschaft (60–80 g/Tag).

##### Gewichtskontrolle

Die empfohlene Gewichtszunahme richtet sich nach dem präkonzeptionellen BMI gemäß den Empfehlungen des Institute of Medicine (Tab. 3; [55]). Eine kurzfristige Gewichtsabnahme von 1–2 kg nach Ernährungsumstellung ist unbedenklich. Regelmäßige Gewichtskontrollen (1-mal/Woche morgens nüchtern zu Hause) werden empfohlen. Eine zu starke oder zu geringe Gewichtszunahme erhöht das Risiko für Komplikationen (z. B. Präeklampsie, fetale Fehlentwicklungen) [30].

**Tab. 3:** IOM-Empfehlungen [55] zur Gewichtszunahme in der Schwangerschaft abhängig von Ausgangs BMI

BMI	BMI-Limits (kg/m^2^) (WHO)	Empfohlene Zunahme während des SS (kg)	Empfohlene Gewichtszunahme/Woche (kg/Woche) (2. + 3. Trimenon)
Untergewicht	< 18,5	13–18	0,5
Normalgewicht	18,5–24,9	11–16	0,4
Übergewicht	25,0–29,9	7–11	0,3
Adipositas	≥ 30,0	5–9	0,2

*BMI* Body Mass Index, *IOM* Institute of Medicine, *SS* Schwangerschaft, *WHO* World Health Organization

#### Körperliche Aktivität

Bewegung in der Schwangerschaft: Bei einer unproblematischen Schwangerschaft ist regelmäßige moderate körperliche Aktivität ein weiterer Bestandteil des Therapiekonzepts. Die Aktivitätszeit sollte dabei mindestens 150 min pro Woche betragen und sollte in den Alltag integriert werden. Bei Ausübung von Sport sollten Sportarten gewählt werden, die mit einer Schwangerschaft vereinbar sind (kein Kontaktsport, Kampfsport, Sportarten mit hoher Sturz- oder Verletzungsgefahr) und dem jeweiligen Trainingszustand entsprechen.

Eine systematische Übersichtsarbeit zeigte bei GDM, dass körperliche Aktivität die Blutzuckerwerte verbessert und den Bedarf an Insulintherapie oder die Insulindosis senken kann [56]. Die Studien zeigten jedoch Unterschiede hinsichtlich Art (Ausdauer‑, Kraft- oder Kombinationstraining) und Dauer der Bewegung (20–50 min/Tag, an 2 bis 7 Tagen/Woche bei moderater Intensität). Es gibt daher keine eindeutige Evidenz, welche Trainingsform den größten Effekt hat.

#### Zusätzliche zu beachtende Faktoren

Bei schwangeren Frauen mit Diabetes und Hypertension sind Blutdruckzielwerte zwischen 110 und 135/85 mm Hg anzustreben [20, 57]. Eine antihypertensive Therapie soll bei Blutdruckwerten > 140/90 mm Hg initiiert werden [58]. Diabetes in der Schwangerschaft ist mit einem erhöhten Präeklampsierisiko verbunden. Daher sollte eine präventive Anwendung von niedrig dosiertem Aspirin (100–150 mg/Tag) zwischen der 12. und 16. Schwangerschaftswoche bei Hypertonie begonnen werden, um die Morbidität, Mortalität und auch Kosten zu senken [20, 59]. In den Leitlinien der Deutschen Diabetesgesellschaft wird eine Beendigung der Therapie bis zur 36. SSW, basierend auf einer randomisiert kontrollierten Studie, zur Verringerung von Blutungsrisiken empfohlen [60, 61].

Diabetische Folgeerkrankungen wie eine Retinopathie, Nephropathie oder autonome Neuropathie können fortschreiten, wobei die Veränderungen meist postpartal reversibel sind und im Langzeitverlauf somit üblicherweise durch die Gravidität selbst keine Progression eintritt. Eine diabetische Retinopathie kann erstmalig in der Schwangerschaft auftreten, aber auch eine Progression in der Schwangerschaft ist möglich, weswegen regelmäßige Kontrollen empfohlen werden (Tab. 4; [20, 62]).

**Tab. 4:** Übersicht: Erforderliche Maßnahmen vor und bei Schwangerschaft bei Frauen mit Typ-1- oder Typ-2-Diabetes mellitus. (Nach [34, 62])

Insulintherapie	Funktionelle Insulintherapie (Basal-Bolus-Prinzip) oder Insulinpumpe werden präferiert
	Der Wechsel auf komplexere Dosierungsformen sollte möglichst vor Beendigung von Verhütungsmethoden erfolgen
Hypoglykämierisiko	Kann limitierend für eine optimale Therapieeinstellung sein
	Besonders in der Frühschwangerschaft bei T1DM ist das Risiko besonders hoch (3- bis 5 × erhöht) [45]
Folsäure	Beginn mit Folsäurepräparat 3 Monate vor Beenden der Verhütung [34]
Augenkontrollen	Kontrolle beim Spezialisten bei Kinderwunsch (Fundus)
	Bei Retinopathie ist, falls erforderlich, eine Therapieeinleitung durchzuführen. Der Kinderwunsch sollte bis zur Stabilisierung einer Retinopathie und Erreichen der Glukoseziele verzögert werden
	Kontrolle: wenn möglich präkonzeptionell/bei Kinderwunsch, jedes Trimester, 3 Monate post partum, danach individuell je nach Erfordernis (mindestens 1‑mal/Jahr)
Nierenfunktion	Stadieneinteilung nach Nephropathieklassifikation (KDIGO)
	Bei Niereninsuffizienz Risikoab- und -aufklärung durch spezialisierte:n Fachärzt:in vor Absetzen der Verhütungsmethoden. Regelmäßige Kontrollen (mindestens 1‑mal/Trimenon) in der Schwangerschaft – Screening auf Albuminurie (Albumin/Kreatinin-Ratio im Harn)
Neurologische Komplikationen	Möglich bei langer Diabetesdauer und schlechter Blutzuckereinstellung
	Evtl. Vorliegen von Hypoglykämiewahrnehmungsstörungen, diabetischer Gastroparese oder orthostatischer Hypotonie
Makrovaskuläre Komplikationen	Schwangerschaft steigert kardiovaskuläres Risiko bei Frauen mit Diabetes
	Erhöhtes Risiko bei: langer Diabetesdauer, höherem Alter, Nikotinkonsum, arterieller Hypertonie, familiärer Hyperlipidämie, positiver Familienanamnese bzw. bei früherem kardiovaskulärem Ereignis, diabetischer Nephropathie
Blutdruck	Zielwert: 110–135/85 mm Hg, Kontraindikation: ACE-Hemmer + AT_1_-Rezeptor-Blocker
	Abklärung einer KHK, wenn vorhanden: Risiko-Ab- und Aufklärung und ggf. Therapieeinleitung
	Erhöhtes Präeklampsierisiko: Acetylsalicylsäure (100–150 mg/Tag) zur Prävention, Beginn bei erhöhtem Risiko vor der 16. Schwangerschaftswoche bis zur 36. Schwangerschaftswoche empfohlen
Lipide	Bempedoinsäure, Ezetimib, PCSK9-Hemmer, Fibrate und Niacin kontraindiziert Einsatz von Statinen während der Schwangerschaft zur Senkung von LDL‑C ist besonderen Fällen vorbehalten, in denen eine Unterbrechung mit eindeutigen Risiken für die Schwangere verbunden ist (z. B. sehr hohes kardiovaskuläres Risiko, familiäre Hypercholesterinämie.) Es muss eine genaue individuelle Aufklärung über mögliche Effekte, Risiken und Nebenwirkungen erfolgen
	Gallesäurebindende Substanzen prinzipiell möglich, aber Nebenwirkungen (Gastrointestinaltrakt) beachten. Schwache Evidenz, in Kasuistiken wurde außerdem ein erhöhtes Risiko von fetalen intrakraniellen Blutungen aufgrund eines Vitamin-K-Mangels beschrieben [121]
Endokrine Abklärung	Messung von TSH und TPO-Antikörpern vor Schwangerschaft
	Bei Übergewicht: Gewichtsreduktion vor Schwangerschaft empfohlen (5–10 %)

*ACE* Angiotensin-Converting-Enzyme, *AT*_*1*_ Angiotensin-II-Rezeptor-Subtyp‑1, *KDIGO* Kidney Disease – Improving Global Outcomes, *KHK* koronare Herzkrankheit, *LDL‑C* Low-density-Lipoprotein-Cholesterin, *PCSK9* Proproteinkonvertase Subtilisin/Kexin Typ 9, *T1DM* Typ-1-Diabetes, *TSH* Thyrotropin, *TPO* Thyreoperoxidase

Frauen mit Nephropathie haben ein deutlich erhöhtes Risiko für die Entwicklung einer Präeklampsie, Frühgeburt sowie eine Wachstumsretardierung des Kindes [62]. Im Falle bereits vor der Schwangerschaft bestehender Spätkomplikationen muss eine Aufklärung der schwangeren Frau über ihr Risiko erfolgen. Während der Gravidität und postpartal sollte eine engmaschige, regelmäßige Beobachtung der Patientin durchgeführt werden (Tab. 4). Eine interdisziplinäre Betreuung in Kooperation mit spezialisierten Nephrolog:innen ist zur Risikoreduktion essenziell. An eine diabetische Neuropathie soll v. a. bei Frauen und Kinderwunsch mit langjähriger Diabetesdauer gedacht werden.

#### Pharmakologische Therapie

Bei unzureichender Einstellung durch Lebensstilmaßnahmen ist unmittelbar eine medikamentöse Therapie bei GDM einzuleiten, bei vorbestehendem Diabetes ist eine Therapieanpassung erforderlich (Abb. 4). Insulin sollte gegenüber oralen glukosesenkenden Medikamenten aufgrund der deutlich besseren Studienlage und keiner Plazentagängigkeit bevorzugt eingesetzt werden [20].

##### Insulin

Sowohl NPH-Insuline als auch lang wirksame Insulinanaloga (Insulin Glargin, Glargin U300, Detemir, Degludec) sind in der Schwangerschaft zugelassen und gelten als sicher. Im Vergleich zu NPH-Insulin zeigen sie keine klaren Vorteile hinsichtlich HbA_1c_ oder Hypoglykämierisiko [50]. Detemir führte in einzelnen Studien zu etwas niedrigeren Nüchternwerten [30]. Schnell wirksame Insuline werden zur Korrektur postprandialer Spitzen angewendet. Bevorzugt werden die kurz wirksamen Insulinanaloga Insulin Aspart und Insulin Lispro verabreicht und mittlerweile in der Regel gegenüber Normalinsulin präferiert eingesetzt. Studien zeigen die sichere Anwendbarkeit von Insulin Lispro und Insulin Aspart in der Schwangerschaft [63, 64]. Für Glulisin liegen in der Gravidität derzeit nur Vigilanzdaten vor [65], die keine besonderen Auffälligkeiten zeigen. Aufgrund der schlechten Datenlage wird eine Anwendung in der Schwangerschaft nicht empfohlen. Die ultraschnell wirksamen Insuline Aspart (Fiasp®) und Lispro (Lyumjev®) sind in der Schwangerschaft ebenso zugelassen. Ultraschnell wirksame Insuline werden rascher resorbiert, sind daher schneller wirksam als bisherige Analoginsuline und werden zur Optimierung postprandialer Hyperglykämien angewendet. Lässt sich die postprandiale Hyperglykämie nicht ausreichend kontrollieren (Glukose 1 h postprandial > 140 mg/dl), kann ein Spritz-Ess-Abstand von 15–30 min vor dem Essen empfohlen werden.

##### Insulinpumpen und AID-Systeme

Beim Einsatz von AID-Systemen bei Frauen mit T1DM sind einige Faktoren zu beachten. Aktuell gibt es zwar keine schwangerschaftsspezifischen Algorithmen, aber 2 in der Schwangerschaft zugelassene Systeme – Medtronic Minimed 780G sowie das CamAPS Fx (YpsoPump plus Dexcom G6- oder Freestyle Libre 3‑CGMS).

Obwohl die Zielglukose im Smartguard nur auf 100 mg/dl gesenkt werden kann, zeigte die CRISTAL-Studie, dass der Einsatz des „advanced hybdrid closed loop systems“ (AHCL, Medtronic Minimed 780G) im Vergleich zu einer Standardinsulintherapie die nächtliche Zeit im Zielbereich, die Zeit im hypoglykämen Bereich sowie die Therapiezufriedenheit in der AHCL-Gruppe verbessert [66]. Bei dem „hybrid closed-loop system“ CamAPS Fx kann ein Glukosezielbereich zwischen 80 und 198 mg/dl eingestellt und somit eine Nüchternglukose von < 95 mg/dl bzw. < 90 mg/dl angestrebt werden. Im Vergleich zur Standardinsulintherapie konnte dieses System einen höheren Prozentsatz der Zeit im maternalen Glukosezielbereich zu allen Zeitpunkten erreichen [67]. Wichtig dabei ist, das mütterliche Gewicht während der Schwangerschaft regelmäßig einzugeben, da das Körpergewicht als Grundlage für den Algorithmus dient. Bei allen AID-Systemen sollen die Bolusfaktoren regelmäßig überprüft und adaptiert werden; gerade das prandiale Glukosemanagement bleibt eine Herausforderung während der Schwangerschaft. Hier werden von Expert:innen sowohl ein Spritz-Ess-Abstand (15–30 min, individuell evtl. länger) und sog. „fake carbs“ (also Kohlenhydrate, die eingegeben aber nicht gegessen werden) empfohlen [68].

Aus aktuellen Studienergebnissen geht hervor, dass auch während der Geburt AID-Systeme eine enge Blutzuckerkontrolle mit Zeit im Zielbereich von 70–82 %, unabhängig von vaginaler Geburt oder Kaiserschnitt, erbringen können [69, 70]. Zielwerte liegen typischerweise bei 70–126 mg/dl (3,9–7,0 mmol/l), um das Risiko für neonatale Hypoglykämie zu senken. Es treten weder vermehrte Hypoglykämien noch Ketoazidosen auf. Bei kritischen Situationen sollte i.v.-Insulin eingesetzt werden.

In den ersten Wochen nach der Geburt bleibt die Zeit im Zielbereich hoch (75–86 %) bei weiterhin niedriger Hypoglykämierate [69, 70]. Stillen erhöht die Insulinsensitivität und das Risiko für Hypoglykämien, die durch AID-Systeme reduziert werden. Der Insulinbedarf sinkt unmittelbar nach der Entbindung deutlich und kehrt erst in den Wochen danach wieder auf Präschwangerschaftsniveau zurück. Einstellungen sollten individuell angepasst und in den ersten Tagen aufgrund von möglichen Schwankungen engmaschig überprüft werden. Eine klinische Checkliste zu AID-Systemen in der Schwangerschaft, während der Geburt und post partum ist in Tab. 5 ersichtlich.

**Tab. 5:** Klinische Checkliste: AID-Systeme während Geburt und post partum [69, 70]

✓ Vorbereitung	Insulininfusionsstelle < 24 h alt, nicht im OP-Bereich (Bauchmitte/suprapubisch)
	Zusätzliche Pumpen- und CGM-Materialien ins Krankenhaus mitbringen (Reservoirs, Sets, Sensor)
✓ Während der Geburt	AID-System mit Schwangerschaftseinstellungen weiterführen (Spontan‑, Einleitungs- oder geplanter Kaiserschnitt)
	Spezifische Systeme: CamAPS FX: Glukoseziel 100–108 mg/dl, Insulin/KH-Ratio 1:12 g–1:15 g Minimed 780 G: Glukoseziel meist 100 mg/dl weiter möglich (falls erforderlich auf 110 oder 120 mg/dl erhöhen), aktives Insulin 2 h, Insulin/KH-Ratio um mindestens 50 % erhöhen
	Blutglukose > 110 mg/dl → Korrekturbolus
	Blutglukose < 70 mg/dl → 8–10 g Kohlenhydrate oral oder i.v. Glukose; ggf. Glukoseziel anpassen (z. B. CamAPS FX „Ease-off“)
	Bei kritischen Situationen oder Unfähigkeit der Patientin, die Pumpe zu bedienen → auf i.v.-Insulin nach Klinikprotokoll umstellen
✓ Nach der Geburt/post partum	Direkt nach Geburt auf Post-partum-Profil umstellen
	Insulinbedarf sofort nach Geburt deutlich niedriger; nächtliche Kontrolle und Stillen berücksichtigen
	Spezifische Systeme: CamAPS FX: Glukoseziel 108 mg/dl, „Ease off“ verwenden je nach Erfordernis, Insulin/KH-Ratio 1:12 g und 1:15 g bei stillenden Müttern Minimed 780 G: Glukoseziel meist 100 mg/dl weiter möglich (falls erforderlich auf 110 oder 120 mg/dl erhöhen), aktives Insulin 2 h, Insulin/KH-Ratio um mindestens 50 % erhöhen (durchschnittlich 80 % bei stillenden Müttern)
	*Pumpeneinstellung für manuelle Bedienung:* *Basalrate*: zwei Drittel der Präschwangerschaftsrate; falls unbekannt, 50 % der Endschwangerschaftsrate oder 0,2–0,25 U/kg/Tag gesamtbasal, stündlich verteilt *Prandial*: 10–20 % schwächer als vor der Schwangerschaft (oder 1:12 g–1:15 g, falls unbekannt) *Korrekturfaktor*: 20 % schwächer als vor der Schwangerschaft (oder 1:50–75 mg/dl)
✓ Abbruchkriterien	Patientin kann Pumpe nicht selbst bedienen (z. B. Komplikationen)
	Anhaltende Hyper- oder Hypoglykämien → Umstellung auf i.v.-Insulin nach Klinikprotokoll; Blutzuckermessung zur Steuerung, CGM nur ergänzend

##### Orale Antidiabetika

Der Sulfonylharnstoff Glibenclamid und das Biguanid Metformin werden in manchen Therapieempfehlungen (z. B. NICE, ADA-Guidelines) als mögliche Alternativen oder zusätzlich zu Insulin in der Schwangerschaft genannt. Die Empfehlung wurde aber zuletzt aufgrund ungewisser Langzeitfolgen bei Nachkommen abgeschwächt [20, 30]. Metformin und Glibenclamid sind plazentagängig. Randomisierte kontrollierte Untersuchungen über den Einsatz von Glibenclamid und Metformin [71, 72] bei GDM zeigten keine wesentlichen Unterschiede zwischen der oralen Behandlung und einer Insulintherapie. Bei Verwendung eines dieser Präparate in der Schwangerschaft sollten die Patientinnen in die Therapieentscheidung miteinbezogen und aufgeklärt werden. Die Verabreichung von anderen oralen und subkutanen glukosesenkenden Medikamenten wie Alpha-Glukosidasehemmer, Glitazone, Glinide, GLP-1-Rezeptoragonisten, DPP-4- und SGLT-2-Hemmer wird in der Schwangerschaft nicht empfohlen. Es fehlt neben den Studiendaten zur sicheren Anwendung auch die Zulassung in der Schwangerschaft.

##### Metformin

An die Gabe von Metformin sollte insbesondere bei übergewichtigen insulinresistenten Frauen als Monotherapie oder in Kombination mit Insulin gedacht werden [34]. Unter Gabe von Metformin ab der 20. Schwangerschaftswoche wurde eine niedrigere Rate schwerer neonataler Hypoglykämien, jedoch eine höhere Frühgeburtenrate beobachtet [71]. Eine Metaanalyse aus 2022 zeigt bei Frauen mit Metformintherapie verglichen zu einer Insulintherapie eine geringere maternale Gewichtszunahme in der Schwangerschaft, weniger Hypoglykämien bei Mutter und Kind sowie ein geringeres Geburtsgewicht [73]. Die Mütter in der Metformingruppe konnten bei der Nachuntersuchung postpartal eher ihr Ausgangsgewicht erreichen als insulinbehandelte Frauen; bezüglich des postpartalen Glukosetoleranzstatus bestanden keine Unterschiede [71]. Ein Grund für einen zögerlichen Einsatz von Metformin ist das Fehlen ausreichender Langzeitdaten zur kindlichen Entwicklung. Die „MiG TOFU“-Studie zeigte, dass Kinder aus der Metformintherapiegruppe in der Schwangerschaft erhöhte subkutane Fettmasse verglichen zur Insulingruppe aufwiesen – die Gesamtkörperfettmasse blieb jedoch vergleichbar [74]. Eine weitere Studie bei Nachkommen von Müttern, die bei PCOS 1700–2000 mg Metformin in der Schwangerschaft erhielten, konnte 4 Jahre nach Entbindung ein deutlich erhöhtes Risiko für Übergewicht und Adipositas im Vergleich zur Placebogruppe feststellen [75]. Die MITY-Studie zeigte bei Metformin-behandelten Schwangeren mit T2DM Vorteile wie bessere Blutzuckerkontrolle, geringere Gewichtszunahme und reduzierten Insulinbedarf [76]. Neugeborene wiesen weniger Adipositas und geringere Größenmaße auf, wodurch die Rate an LGA-Neugeborenen sank, jedoch stieg die Zahl der SGA-Kinder. Die langfristigen Auswirkungen auf die Kinder sind noch unklar und sollten in der Beratung berücksichtigt werden.

Bei Frauen mit PCOS und Metformintherapie zu Beginn der Schwangerschaft zur Ovulationsstimulierung wird eine Beendigung der Metformintherapie vor Ende des ersten Trimesters empfohlen [20]. Unter einer Therapie mit Metformin bei schwangeren Frauen mit PCOS wurde über eine erhöhte Prävalenz von Übergewicht/Adipositas bei den Kindern im Alter von 4 Jahren berichtet [75].

##### Sulfonylharnstoff

Die Anwendung von Sulfonylharnstoffen wird in der Schwangerschaft nicht empfohlen. Wird eine notwendige Insulintherapie von der Patientin abgelehnt, kann Glibenclamid als selten genutzte Alternative zur Behandlung des Gestationsdiabetes (GDM) in Betracht gezogen werden.

Allerdings zeigen Studien klare Nachteile: In einer Metaanalyse war Glibenclamid mit einer stärkeren maternalen Gewichtszunahme sowie höheren Raten an fetaler Makrosomie und neonataler Hypoglykämie im Vergleich zu Metformin verbunden [77]. Auch im Vergleich zu Insulin traten unter Glibenclamid häufiger Makrosomien, neonatale Hypoglykämien und ein höheres Geburtsgewicht auf [77]. In einer randomisiert kontrollierten Studie zeigten sich unter Glibenclamid zudem häufiger kumulative perinatale Komplikationen, darunter Makrosomie, Hypoglykämie und Hyperbilirubinämie, im Vergleich zu Insulin [78]. Auch wenn in dieser Studie keine signifikant erhöhte Makrosomierate unter Glibenclamid im Vergleich zu Insulin beobachtet wurde, war die Hypoglykämierate bei durchschnittlich niedriger Glibenclamid-Dosis (5,4 mg/Tag) signifikant höher als unter Insulin.

#### Geburtshilfliche Überwachung

Schwangere mit Diabetes in der Schwangerschaft sollten in einem Krankenhaus mit diabetologischer Erfahrung und angeschlossener Neonatologie entbunden werden. Empfohlen werden:

- 1- bis 3‑wöchentliche klinische Kontrollen,
- bei Hyperglykämie in Frühschwangerschaft: frühes Organscreening durch Ultraschall zum Ausschluss von Fehlbildungen (v. a. Herz, Niere),
- Ultraschall (Biometrie, Fruchtwasser, evtl. Doppler), Wachstumskurven (v. a. Wachstumszunahme des Abdomens = asymmetrische Wachstumszunahme; Polyhydramnion) beachten,
- Achten auf erhöhtes Risiko zur Entwicklung einer Schwangerschaftshypertonie, Präeklampsie, Infektionen,
- idealen Geburtstermin und Geburtsmodus festlegen.

Schwangere Frauen, die einen GDM im Laufe der Schwangerschaft entwickeln, zeigen eine herabgesetzte Insulinsensitivität bereits vor der Schwangerschaft [79, 80]. Dies könnte zu oxidativem Stress in der ersten Phase der Schwangerschaft führen und eine Ursache für kongenitale Defekte darstellen [81]. Um große anatomische Fehlbildung zu erkennen, ist für jede schwangere Frau ein Screening für Fehlbildungen und Chromosomenstörungen sowie für Präeklampsie im 1. Trimenon (Schwangerschaftswoche 11 bis 13 + 6) empfohlen. Zusätzlich wird ein Organscreening im 2. Trimenon (Schwangerschaftswoche 19 + 0 bis 22 + 6) empfohlen [60, 82]. Da die mütterliche Hyperglykämie einen direkten Einfluss auf die fetale Hyperglykämie, Hyperinsulinämie und letztendlich auf das fetale Wachstum hat [83], sind sonographische fetale Wachstumskontrollen alle 2 bis 4 Wochen empfohlen [84]. Regelmäßig durchgeführte Ultraschallkontrollen führen zu einem besseren neonatalen Outcome und sollen zu einem nicht ultraschallbasierten Management präferiert werden [85]. Hierbei soll das erwartete fetale Gewicht (EFW) durch die Vermessung vom Kopfumfang (KU), Abdomenumfang (AU) und Femurlänge (FL) geschätzt werden [86]. Unter den geburtshilflichen Komplikationen eines GDMs erkennt man die fetale Makrosomie, welche bereits ab der 24. Schwangerschaftswoche sonographisch diagnostiziert werden kann, wenn der Abdomenumfang (AU) eine akzelerierte Wachstumstendenz aufweist [87]. Eine übermäßige Fruchtwassermenge (Polyhydramnion) wird als Hinweis einer diabetischen Fetopathie gesehen, wobei bis dato keine Referenzwerte festgelegt wurden [60]. Die Messung des fetalen subkutanen Fettgewebes könnte als Zusatzparameter für die Evaluation der diabetischen Fetopathie herangezogen werden, dies ist aber heute aufgrund der mäßigen Reproduzierbarkeit der Messwerte noch nicht Teil der Routineuntersuchungen bei GDM-Schwangerschaften [88]. Eine Überschreitung des Geburtstermins sollte bei Schwangeren mit insulinpflichtigem GDM vermieden werden. Ob zwischen Schwangerschaftswoche 38 + 0 und 40 + 0 eine Geburtseinleitung stattfinden soll, soll individuell entschieden werden. Dabei sollen der Insulinbedarf, die Ultraschallbefunde (Kindsgewicht, Doppler, Fruchtwasser), maternale Erkrankungen wie Präeklampsie und die vorausgegangenen Schwangerschaftsverläufe in die Entscheidung miteinbezogen werden [89–91]. Eine Einleitung wegen schlechter Blutzuckereinstellung vor Schwangerschaftswoche 38 + 0 sollte wegen Frühgeburtlichkeit-bedingter Morbidität vermieden werden. Vielmehr sollte eine pränatale Optimierung der Blutzuckerwerte erfolgen. Es ist bekannt, dass das Risiko für eine Schulterdystokie ab einem Geburtsgewicht von 4250 g signifikant ansteigt [92]. Ab einem geschätzten Geburtsgewicht von 4500 g sollte deshalb bei einer Schwangeren mit GDM eine Sectio empfohlen werden. Bei einem Schätzgewicht von 4000–4499 g sollte eine differenzierte Aufklärung der Schwangeren über individuell erhöhtes Schulterdystokierisiko erfolgen, insbesondere bei ausgeprägter Kopf-Abdomen-Differenz.

#### Überwachung und Management des Neugeborenen (Abb. 5)

Ein Routinemonitoring ist für eine Hochrisikopopulation an Neugeborenen sinnvoll, zu denen Kinder aus diabetischen Schwangerschaften bzw. solche, die aus einem anderen Grund einem erhöhten Risiko für die Entwicklung einer Hypoglykämie ausgesetzt sind, zählen (z. B. dystrophe Neugeborene; LGA-Babys). Generell zu vermeiden sind prolongierte und rezidivierende Hypoglykämien, da diese mit akuten systemischen und langfristigen neurologischen Konsequenzen einhergehen können [93].

**Abb. 5:**
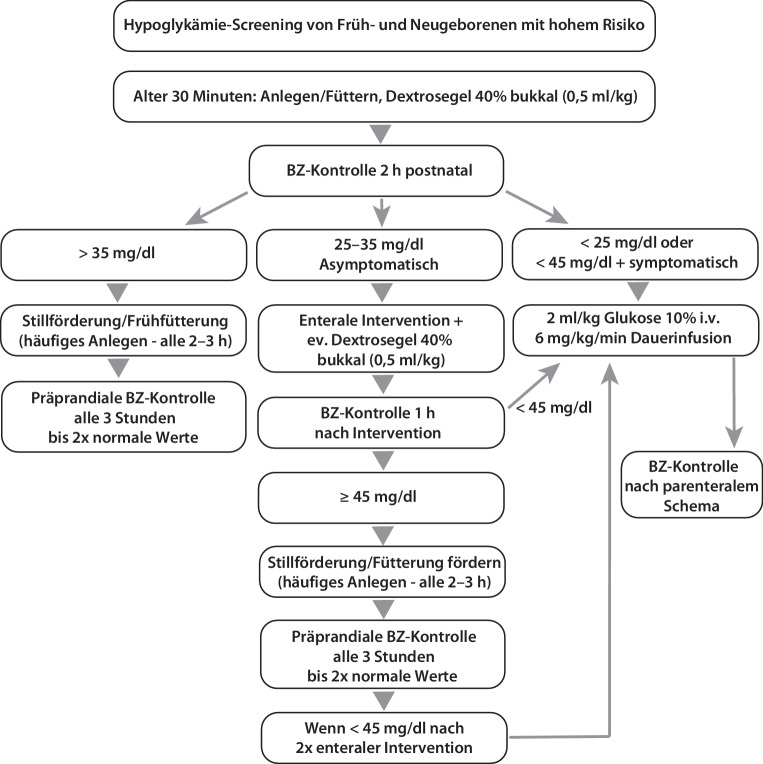
Flussdiagramm Hypoglykämiescreening von Früh- und Neugeborenen mit hohem Risiko. *BZ* Blutzucker, *i.v.* intravenös

##### Peripartales Management und Blutglukosebestimmungen nach Geburt

Der präventive Ansatz sieht vor, dass Neugeborene mit einem hohen Hypoglykämierisiko in den ersten 30 Lebensminuten Nahrung erhalten sollen, am besten durch direktes und prolongiertes Anlegen (~20 min) [94]. Dieses Vorgehen ist nach Kaiserschnittentbindung möglich und ist auch die effektivste Intervention, um den Blutzucker zu stabilisieren und Hypoglykämien zu reduzieren [95, 96]. Dabei ist „skin-to-skin care“ zwischen Mutter und Kind essenziell, um das Hypothermierisiko zu senken (reduziert Energieverbrauch und erleichtert die Aufrechterhaltung eines normalen Blutzuckerspiegels), während das Stillen und die Milchproduktion gefördert werden [97–100]. Bei hohem Hypoglykämierisiko (z. B.: insulinpflichtiger GDM) ist eine 1‑malige bukkale Gabe von 40 % Glukosegel (0,5 ml/kg) 45–60 min nach der Geburt zu erwägen [101, 102]. Die Gabe von bukkalem 40 % Glukosegel zeigte sich sicher und effektiv. Vor allem in Kombination mit Stillen bzw. frisch gewonnenem Kolostrum (0,5 ml/kg Kolostrum vs. 4,5 ml/kg Formula) konnte der Blutzucker optimal stabilisiert werden [103, 104].

Ist ein frühzeitiges Anlegen nicht möglich bzw. von der betreuenden Pflege/Hebamme das Anlegen für insuffizient befundet, soll das Kind pränatal gewonnenes Kolostrum (frisch vor Sectio – 0,5 ml/kg), pasteurisierte Frauenmilch oder Formula-Nahrung (3–5 ml/kg) erhalten. Weitere Blutzuckermessungen nach peripartaler Prophylaxe: Zumindest 2‑mal vor den nächsten beiden Mahlzeiten (ca. nach 3 und 6 h, evtl. auch nach 12 h z. B. bei mütterlichem Diabetes, grenzwertigen Messungen). Ende der Messungen: Es sollen zumindest 2 normale präprandiale Glukosewerte hintereinander dokumentiert sein, um die Messungen beenden zu können. Messung nach Intervention: Bei enteraler oder intravenöser Intervention aufgrund einer Hypoglykämie erfolgt eine Kontrolle 1 h nach Intervention. Die Bestimmung der Blutglukose muss unmittelbar nach der Blutabnahme erfolgen. Bei Verwendung von Schnelltests (Glukometer) weisen diese im hypoglykämischen Bereich unter 45 mg/dl Glukose in Abhängigkeit vom Hersteller Ungenauigkeiten auf. Ein mit dieser Messmethode ermittelter hypoglykämischer Wert soll durch eine laborchemische Bestimmung kontrolliert werden. Dies sollte aber zu keiner Verzögerung der Therapie führen.

##### Interventionsgrenzen und therapeutische Zielwerte

Aufgrund interindividueller Schwankungen gibt es keine absoluten Grenzwerte für die Behandlung der Hypoglykämie des Neugeborenen. Vorgeschlagen werden pragmatische „Interventionsgrenzen“, bei denen eine Intervention in Erwägung gezogen werden sollte (s. Intervention: < 25 mg/dl intravenös, 25–35 mg/dl enteral). Die „therapeutischen Zielwerte“ beinhalten einen Sicherheitsabstand.

##### Ernährung des Säuglings nach Geburt

Das Abpumpen von Kolostrum vor der Geburt kann bei Schwangeren mit Diabetes in der Schwangerschaft vorteilhaft sein, um die neonatale Hypoglykämie zu bekämpfen bzw. zur Reduktion der Notwendigkeit von Formula-Nahrung und zur Förderung des frühen Stillbeginns und der Milchbildung [105]. Neugeborene sollen bereits innerhalb der ersten Lebensstunde angelegt werden, dies gilt besonders für Kinder aus diabetischer Schwangerschaft. Stillen senkt das Risiko für späteres Übergewicht des Kindes, das gilt v. a. für Kinder von Müttern mit Gestationsdiabetes [106–108]. Vor allem in dieser Kohorte hat Stillen einen hohen protektiven Stellenwert, weil Kinder von Müttern mit Gestationsdiabetes im Erwachsenenalter ein erhöhtes Risiko für eine gestörte Glukosetoleranz haben [109]. Dennoch ist die Stillrate bei Frauen mit Diabetes (Typ I und Typ II) deutlich niedriger und die Stilldauer kürzer [110, 111]. Aus diesem Grund sollte bereits vor der Entbindung ausdrücklich eine Stillförderung stattfinden, und die Frauen sollten ermutigt werden, ihre Kinder zu stillen [112]. Nahrung aus der Flasche (Anfangsmilch) soll nur angeboten werden, wenn Stillen nicht möglich/erwünscht ist bzw. als Intervention bei zu niedrigem Blutzucker (s. „Intervention“) [95].

##### Intervention

Enteral: Nur bei asymptomatischer Hypoglykämie 25–35 mg/dl → Verabreichung von 10–20 ml (3–5 ml/kg) pränatal gewonnenes Kolostrum, Frauenmilch oder Formula-Nahrung. Alternativ ist die Verwendung von bukkal zu verabreichendem 40 %igem Dextrosegel möglich. Intravenös: Bei deutlicher Hypoglykämie < 25 mg/dl, symptomatischen Kindern < 45 mg/dl oder persistierender Hypoglykämie (falls die Kontrolle 1 h nach Intervention < 45 mg/dl ist oder falls trotz 2‑maliger enteraler Intervention weiter korrekturbedürftige präprandiale Blutzuckerwerte gemessen werden) → 2 ml/kg Glukose 10 % als i.v.-Bolus, gefolgt von 6–8 mg/kg/min Glukose als kontinuierliche Infusion. Es wird eine schrittweise Reduktion der intravenösen Glukosezufuhr unter Beginn der enteralen Ernährung und präprandialen Blutzuckerkontrolle empfohlen. Kinder von Frauen mit GDM haben ein höheres Risiko, im späteren Leben übergewichtig zu werden und ein metabolisches Syndrom bis hin zu einem Diabetes zu entwickeln [113]. Deshalb sollte bei allen – und besonders bei makrosomen – Kindern auf eine normale Gewichtsentwicklung geachtet werden (s. „Nachbetreuung der Kinder“).

## Nachbetreuung der Mutter bei Diabetes in der Schwangerschaft

Nach Entbindung sind eine rasche Reduktion der Insulindosen um etwa 50 % und enge Blutzuckerkontrolle erforderlich, da die Insulinsensitivität rasch zunimmt [20]. Bei Frauen mit T1DM oder T2DM sind weitere regelmäßige Blutzuckerkontrollen zu empfehlen. Falls bei Frauen mit Gestationsdiabetes nach der Geburt normale Blutzuckerwerte erhoben werden (nüchtern < 100 mg/dl und unabhängig von Mahlzeiteneinnahme postprandial < 200 mg/dl), ist keine weitere definierte Ernährungstherapie oder Blutzuckerselbstmessung notwendig.

Allerdings muss bei GDM 4 bis 12 Wochen nach der Geburt eine Reklassifizierung der mütterlichen Glukosetoleranz mittels Standard-oGTT erfolgen. Bei pathologischem Befund nach den allgemeinen Grenzwerten zur Diabetesklassifikation müssen Therapieempfehlungen erfolgen (s. Leitlinien Diabetes mellitus – Definition, Klassifikation, Diagnose, Screening und Prävention [2026] und Antihyperglykämische Therapie bei Diabetes mellitus Typ 2). Im Fall eines postpartal bestehenden Prädiabetes (gestörte Glukosetoleranz (2-h-Wert 140–199 mg/dl) im oGTT oder erhöhter Nüchternglukose (100–125 mg/dl)) wird eine Lebensstiländerung mit Ernährungs- und Bewegungsberatung empfohlen.

Eine Subanalyse des Diabetes Prevention Programs zeigte, dass bei vergleichbarer Ausgangslage bezüglich Glukosetoleranzstatus und Insulinresistenz Frauen mit GDM-Anamnese ein doppelt so hohes Risiko für die Progression zu einem manifesten Diabetes aufwiesen wie jene, die eine unauffällige Schwangerschaft hatten. Weiters profitierte diese Gruppe von einer Therapie mit Metformin besonders [114]. Dies wurde im 10-Jahres-Follow-up erneut bestätigt: Lebensstilmaßnahmen und Metformin konnten das Diabetesrisiko um 35–40 % verglichen zu Placebo verringern [115]. Eine Analyse des Wiener GDM-Programms zeigte, dass ein 2‑h-Blutzuckerwert im ersten oGTT post partum über 140 mg/dl, ein HDL unter 50 mg/dl und ein Alter über 35 Jahre die wichtigsten unabhängigen Risikofaktoren für die Entwicklung eines manifesten Diabetes innerhalb von 10 Jahren darstellten [116]. Untersuchungen belegen nun auch für die seit einigen Jahren geltende GDM-Diagnoserichtlinie (basierend auf der HAPO-Studie) ein mehr als 3‑fach höheres Risiko für eine Glukosestoffwechselstörung bei Frauen mit GDM im Vergleich zu Frauen mit normaler Glukosetoleranz nach 11 Jahren Follow-up [117]. Entsprechend der Datenlage müssen alle Frauen mit GDM außerdem über ihr erhöhtes Risiko für die Entwicklung eines T2DM, eines GDM-Rezidivs (20–50 %) bei neuerlicher Schwangerschaft, ein erhöhtes kardiovaskuläres Risiko sowie über Möglichkeiten der Diabetesprävention informiert werden [117]. Frauen mit Diagnose eines frühen GDM haben dabei ein besonders hohes Risiko für die Entwicklung eines T2DM [118]. Bei unauffälligem Erstbefund sollen die Frauen alle 1 bis 3 Jahre mittels oGTT oder zumindest mittels Messung der Nüchternglukose und des HbA_1c_ nachuntersucht werden. Frauen mit Diabetes in der Schwangerschaft sollen, wenn immer es möglich ist, ihr Kind stillen, da protektive Effekte in Studien gezeigt werden konnten [20, 34]. Bei einer Stilldauer von mehr als 3 Monaten weisen stillende Mütter eine um bis zu 10 Jahre verzögerte Progression von GDM zu T2DM auf als nicht stillende Frauen [119].

## Nachbetreuung der Kinder von Müttern mit Hyperglykämie in der Schwangerschaft

Bei Nachkommen von GDM-Schwangerschaften ist ein erhöhtes Risiko für Übergewicht/Adipositas und T2DM bekannt [117, 120]. Ein gesunder Lebensstil und regelmäßige Gewichtskontrollen sind zu empfehlen. Bei Hinweisen auf Hyperglykämie ist eine sofortige Abklärung empfohlen (s. auch Leitlinie Diabetes mellitus – Definition, Klassifikation, Diagnose, Screening und Prävention). Ein T2DM-Screening sollte bei asymptomatischen Kindern und Jugendlichen bei Adipositas (BMI > 95. Perzentile, geschlechts- und altersadjustiert) oder Übergewicht (BMI > 85. Perzentile) und mütterlichem GDM in der Schwangerschaft des Kindes erfolgen [41].

## Interessenkonflikt

A. Kautzky-Willer hat von folgenden Unternehmen, die teils auch fördernde Mitglieder der ÖDG sind, Forschungsunterstützungen und/oder Honorare erhalten: AstraZeneca, Bristol-Myers Squibb, GlaxoSmithKline Pharma, Eli Lilly, Merck Sharp & Dohme, Novartis, Novo Nordisk, Roche, Sanofi-Aventis. Y. Winhofer hat von folgenden Unternehmen, die auch fördernde Mitglieder der ÖDG sind, Forschungsunterstützungen/Honorare erhalten: Abbott, Dexcom, Eli Lilly, Novo Nordisk, Roche, Sanofi-Aventis. M. Leutner hat von folgenden Unternehmen, die auch tlw. fördernde Mitglieder der ÖDG sind, Forschungsunterstützungen und/oder Honorare erhalten: Abbott, AstraZeneca, Bayer, Boehringer Ingelheim, Daiichi Sankyo, Novartis, Novo Nordisk, Ultragenyx. A. Berger hat von folgenden Unternehmen Vortragshonorare und Forschungsunterstützung erhalten: AstraZeneca, Sanofi, Roche, Merck Sharp & Dohme, Chiesi und Prolacta. R. Weitgasser hat von folgenden Unternehmen, die teils auch fördernde Mitglieder der ÖDG sind, Forschungsunterstützungen und/oder Honorare erhalten: Abbott Diabetes Care, Amgen, Boehringer Ingelheim, Daiichi Sankyo, Eli Lilly, Novo Nordisk, Sanofi. J. Harreiter hat von folgenden Unternehmen Forschungsunterstützungen und/oder Honorare erhalten: Amgen, AstraZeneca, Bayer, Boehringer Ingelheim, Novartis, Novo Nordisk, Ultragenyx. H. Kiss, V. Falcone, T. Linder, L. Wisgrill, M. Lechleitner und S. Huber geben an, dass kein Interessenkonflikt besteht.
